# Superior Enhancement of the UHMWPE Fiber/Epoxy Interface through the Combination of Plasma Treatment and Polypyrrole In-Situ Grown Fibers

**DOI:** 10.3390/polym15102265

**Published:** 2023-05-11

**Authors:** Xiaoning Yang, Zhongwei Zhang, Yuhang Xiang, Qingya Sun, Yilu Xia, Ziming Xiong

**Affiliations:** 1 School of Mechanical Engineering, Nanjing University of Science & Technology, Nanjing 210094, China; 2 State Key Laboratory of Explosion & Impact and Disaster Prevention & Mitigation, Army Engineering University of PLA, Nanjing 210007, China

**Keywords:** UHMWPE fiber, plasma treatment, polypyrrole, interfacial performance

## Abstract

Obtaining a robust fiber/matrix interface is crucial for enhancing the mechanical performance of fiber-reinforced composites. This study addresses the issue by presenting a novel physical–chemical modification method to improve the interfacial property of an ultra-high molecular weight polyethylene (UHMWPE) fiber and epoxy resin. The UHMWPE fiber was successfully grafted with polypyrrole (PPy) for the first time after a plasma treatment in an atmosphere of mixed oxygen and nitrogen. The results demonstrated that the maximum value of the interfacial shear strength (IFSS) of the UHMWPE fiber/epoxy reached 15.75 MPa, which was significantly enhanced by 357% compared to the pristine UHMWPE fiber. Meanwhile, the tensile strength of the UHMWPE fiber was only slightly reduced by 7.3%, which was furtherly verified by the Weibull distribution analysis. The surface morphology and structure of the PPy in-situ grown UHMWPE fibers were studied using SEM, FTIR, and contact angle measurement. The results showed that the enhancement of the interfacial performance was attributed to the increased fiber surface roughness and in-situ grown groups, which improved the surface wettability between the UHMWPE fibers and epoxy resins.

## 1. Introduction

An ultra-high molecular weight polyethylene (UHMWPE) fiber is characterized by high strength, low density, and outstanding toughness [1,2,3]. Nowadays, it has played a vital role in individual protection, armor protection, and other essential areas [4,5]. However, the poor interfacial bonding strength between UHMWPE fibers and epoxy resin has led to its limitations in engineering applications. The main reason is that the fiber surface is smooth with no polar groups [6,7,8]. Therefore, developing effective fiber surface treating methods to improve the property of the UHMWPE fiber/epoxy interface has become a top priority.

The main methods have been presented by introducing polar functional groups or increasing the fiber surface roughness to improve the interfacial bonding, including the chemical, physical, and physical–chemical process methods [9,10,11]. The chemical method includes strong acid oxidation, grafting, organic coating, etc. By incorporating -OH, -CO, and -C=O groups onto the surfaces of the fibers, their polarity is enhanced, resulting in improved wetting capabilities [12]. Wang et al. [13] coated the UHMWPE with tannic acid (TA)-Na^+^ to improve the wettability and adhesion between the fiber and the resin, showing that the interfacial shear strength increased by 43.3%. Meanwhile, Han et al. [14] utilized a two-step PVA-glutaraldehyde condensation (Corona-PG-2S) method, enhancing the peel strength by 262.8%. However, this method usually leads to a decrease in the tensile strength of the fiber. In addition, chemical treatment methods could also cause environmental pollution.

In comparison, the physical method has a milder effect on the interface modification, environmental pollution, damage to the fiber, and impact on the properties of the material itself [15]. The commonly used physical methods are plasma treatment and corona discharge [16,17]. By performing plasma ionization treatment under different atmospheres, the fiber surface can be etched with increasing surface roughness, and the chemical structure of the fiber surface can be changed as well. For example, the UHMWPE fiber treated with air plasma for 4 s can increase the interfacial bonding strength of the epoxy resin by 84% [18]. However, the modification effect of the physical method usually decays rapidly with the increased storage time [19,20].

In general, relying solely on the physical or chemical method usually leads to a decrease in the fiber strength, considerable environmental pollution, or a quick reduction in the modification effectiveness. Recently, combining these two methods became a viable and practical approach for the surface treatment. For example, Cech et al. [21] initially subjected the glass fiber to an oxygen plasma treatment and subsequently carried out chemical grafting of tetravinylsilane. This approach enhanced the fiber’s interfacial adhesion by augmenting its surface roughness and introducing polar groups without impairing the fiber structure. Additionally, Kelsey and Henry [22] reported the combination of the plasma treatment and ZnO NWs grafting methods on the UHMWPE fiber and found that the interfacial shear strength (IFSS) was increased by 135% compared to the pristine fiber. In addition, Zhu et al. [23] found that the combined use of plasma and Poly (p-phenylene terephthalamide) treatments could achieve a 91% increase in the IFSS of the UHMWPE fibers, which was superior to the sole use of the plasma treatment method.

To improve the interface performance between the UHMWPE fiber and the epoxy matrix, a physical–chemical synergistic modification method was presented in this study. The UHMWPE fiber was successfully grafted with polypyrrole (PPy) for the first time after the plasma treatment in an atmosphere of mixed oxygen and nitrogen. The synergy effects of the plasma treatment and in-situ grown PPy on the UHMWPE fiber/epoxy interface were systematically studied using contact angle measurements, infrared spectroscopy (FTIR), a scanning electron microscope (SEM), and mechanical property testing. Through these means, it was promising to significantly enhance the IFSS of the UHMWPE fiber/epoxy without dramatically sacrificing the tensile strength of the UHMWPE fiber. The research results are expected to provide a high-efficiency, economical, and environmentally friendly treatment method for the surface modification of the UHMWPE fiber, and then offer ideas for improving the mechanical properties of the UHMWPE fiber-reinforced composites.

## 2. Experimental Details

### 2.1. Materials

The UHMWPE fibers used were provided by China Yixing Xinli Weaving Co., Ltd. (Yixing, China) The PPy was prepared by means of a concentration of 40 m mol/L of pyridide (C4H5N, Shanghai Shengen Chemical Technology Co., LTD., Shanghai, China) and ammonium persulfate ((NH4)2S2O8, analytical grade, Zhenjiang Jiuyi Chemical Reagent Co., LTD., Zhenjiang, China).

The fibers were processed using different treatment methods with the IN2 infusion-type low-viscosity epoxy (provided by the Easy Composites company, London, United Kingdom) resin as the matrix and AT 30 as the curing agent with a mixing mass ratio of 10:3.

### 2.2. Surface Treatments

A sub-atmospheric pressure glow discharge processor (HPD-2400, Suman Plasma Technology Limited Nanjing, Nanjing, China) was applied to the UHMWPE fibers with a cavity size of 720 (W) mm × 1360 (H) mm × 765 (D) mm. The power was set to 350 w. The UHMWPE fibers were plasma-treated under three different atmospheres, including N_2_:O_2_ = 5:5, N_2_:O_2_ = 8:2, and pure N_2_.

**Figure 1:**
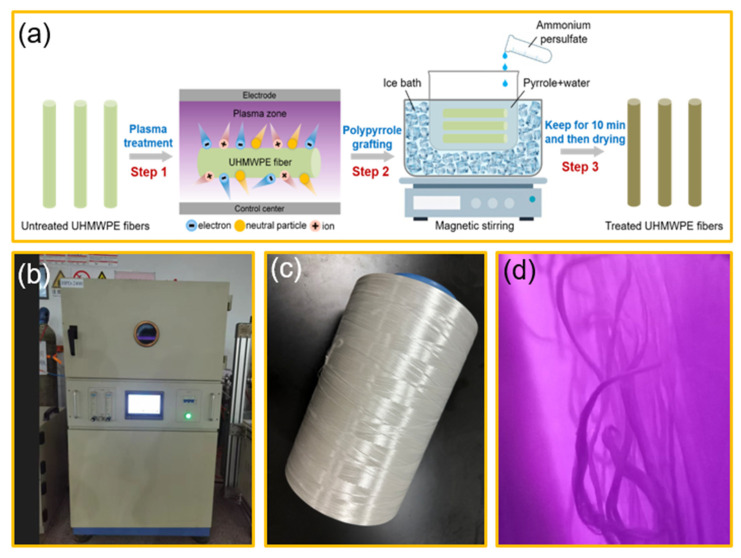
(**a**) Plasma–PPy synergistic treatment process; (**b**) plasma processing equipment; (**c**) utilized UHMWPE fibers; (**d**) plasma-treated UHMWPE fibers.

The fibers before and after the plasma treatment were grafted with PPy. The specific process was as follows. First, the samples were placed in the pyrrole aqueous solution and stirred using a magnetic stirrer covered with ice for 10 min. Secondly, the ammonium persulfate was dropped slowly into the above solution and then stir for an additional 30 min. Finally, the samples were taken out and dried in an oven. The plasma–PPy synergistic treatment process is shown in Figure 1a. The photograph containing the plasma processing equipment and the UHMWPE fibers are shown in Figure 1b–d, respectively. The sample specifications before and after the surface treatment are listed in Table 1.

### 2.3. Mechanical Performance Test

**Figure 2:**
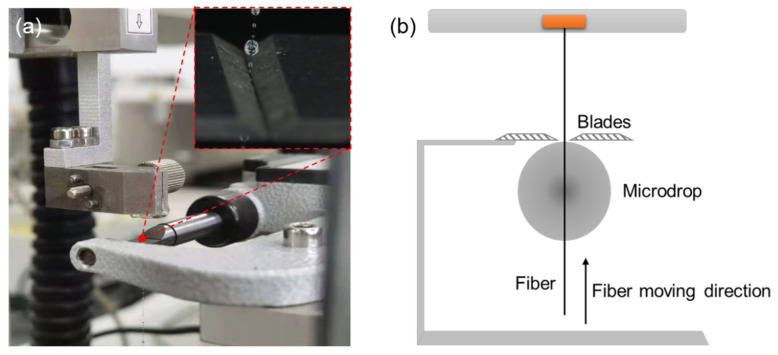
(**a**) Photo of the pull-out test device; (**b**) Schematic diagram of the pull-out test.

The microsphere debonding method was used for analyzing the bonding performance of the fiber and the matrix interface. Double-sided tape was used to fix the UHMWPE fiber monofilament on the fixture frame. A fine needle was utilized to dip a small amount of the configured resin-curing agent system (mass ratio 100:30) on the fiber surface. To obtain a microsphere debonding test sample, the resin droplets were solidified according to the same curing process parameters for 6 h under 60 °C. Ten microspheres were selected with the same size and shape as in one group of testing. The YG163 drawing tester (Wenzhou Jigao Testing Instrument Co., Ltd., Wenzhou, China) was used to test the bonding performance of the fiber/matrix interface, performing at a 0.2 mm/min loading speed. Figure 2 shows the photo and schematic diagram of the pull-out test device. The interfacial shear strength *τ_d_* (IFSS) was calculated using Equation (1).

(1) $$\tau_{d}=F_{\max}/d\pi l_{e}$$

where *F_max_* is the maximum load (N) when the resin is debonded, *d* is the fiber diameter (m), and *l_e_* is the embedding length (m).

The UHMWPE fiber monofilament tensile test was performed using the FAVIMAT+ fiber tester (Lianyungang Fiber New Material Research Institute Co., Ltd., Lianyungang, China) according to the standard GB/T 31290-2014 Carbon fiber Determination of the tensile properties of single-filament specimens with a tensile rate of 2.0 mm/min. Each reported tensile test value was the average of more than ten successful measurements.

### 2.4. Characterization Methods

The Quanta 250FEG scanning electron microscope (SEM) was used to observe the microscopic morphology of the samples before and after the surface treatment. The voltages were 5 KV and 20 KV. The sample surface was treated using plasma sputtering equipment to spray gold to improve its conductivity. The functional groups on the surface of the fibers were analyzed using Fourier transform infrared spectroscopy (FTIR). In the test, the spectrometer’s resolution was set to 4000, and the wavenumber range was 400–4000 cm^−1^.

The surface tension of the liquid epoxy resin fibers was measured using the static drop method, and the wettability of the fibers before and after the modification was analyzed. The contact angle was calculated according to Equation (2).

(2) $$\theta /2=\tan^{-1}\left(2h/w\right)$$

where *h* is the height of the drop on the surface of the fiber, *w* is the width of the drop on the surface of the fiber, and *θ* is the contact angle.

## 3. Results and Discussion

### 3.1. Microsphere Debonding Tests

**Figure 3:**
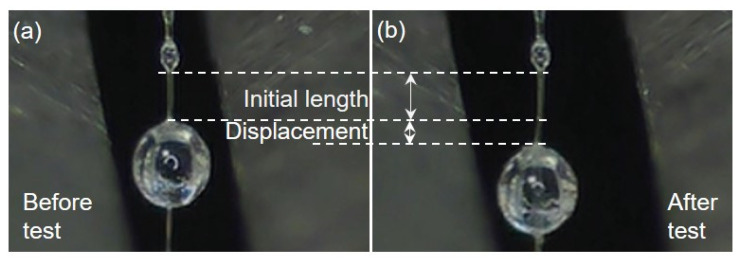
(**a**) The distances of the microspheres before microsphere debonding test; (**b**) The distances of the microspheres fter microsphere debonding test.

**Figure 4:**
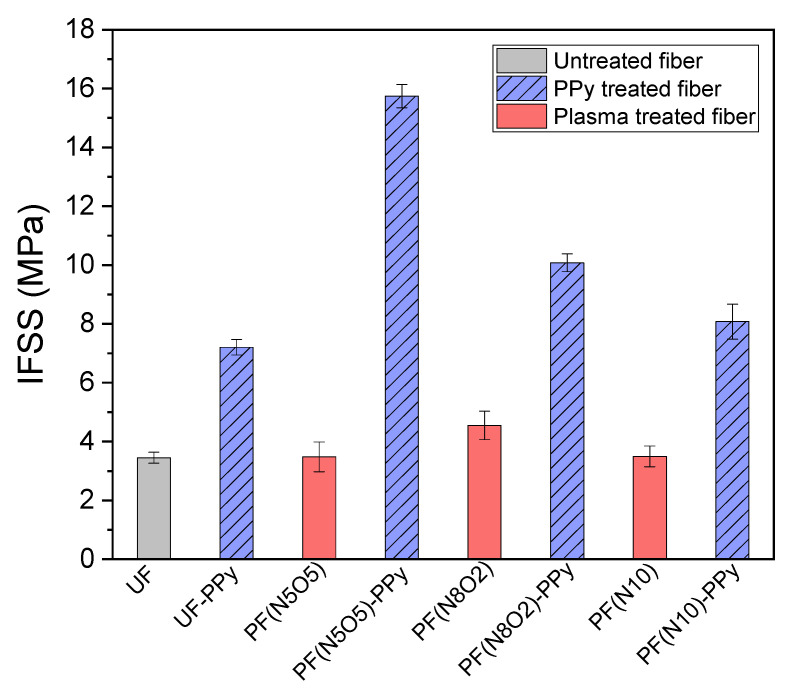
Debonding test results.

The changes in the two microspheres before and after the microsphere debonding test are shown in Figure 3. It was found that the position of the microsphere moved downward after the test, and the morphology of the microsphere was undamaged. This meant that during the pull-out test, only the shear failure mode was found, making it possible to obtain accurate interfacial shear strength data, as listed in Figure 4.

The debonding test results of the microspheres under the different treatment processes are shown in Figure 4. It can be seen that the interface shear strength of all the UHMWPE fiber samples subjected to the plasma treatment was higher than that of the untreated fibers. Among them, the interfacial shear strength of the sample PF(N5O5) treated using plasma with the mixed gas was 0.89% higher than that of the untreated fiber, and the sample of PF(N8O2) had the most considerable interface shear strength, which was 32% higher than that of the untreated fiber. The sample PF(N10) treated using pure nitrogen plasma was 1.5% higher than the untreated fiber. This illustrates that the mixed gas plasma treatment process can increase the fiber/matrix interfacial adhesion performance with limited improvement. When the plasma-treated UHMWPE fibers were grafted with PPy, the interfacial shear strength was significantly enhanced. It can be found that the interfacial shear strength of the fibers PF(N5O5)-PPy, PF(N8O2)-PPy, PF(N10)-PPy were increased by 357%, 192%, and 134%, respectively, in which the UHMWPE fiber PF(N5O5)-PPy had an excellent interfacial adhesion performance. This indicated that the UHMWPE fibers surface grafted with PPy could effectively improve the interfacial adhesion performance. Nevertheless, the interfacial shear strength of the untreated fiber was lower than the other fibers grafted with PPy.

Furthermore, by comparing the interfacial shear strength of the fiber before and after the PPy grafting under the same plasma treatment atmosphere, the interfacial shear strength of the fiber was significantly increased by between 109–353%. Among them, the PF(N5O5)-PPy had the most significant increase, reaching 353%. In general, both the single experimental method and the method of synergistic treatment can improve the interfacial adhesion performance. It should be noted that the synergistic approach resulted in a noteworthy improvement in the IFSS. The process of the surface treatment using PPy grafting and plasma treatment with the mixture of oxygen and nitrogen achieved the highest increase in the IFSS, which showed a 357% higher enhancement than that of the pristine fibers.

### 3.2. Tensile Properties

**Figure 5:**
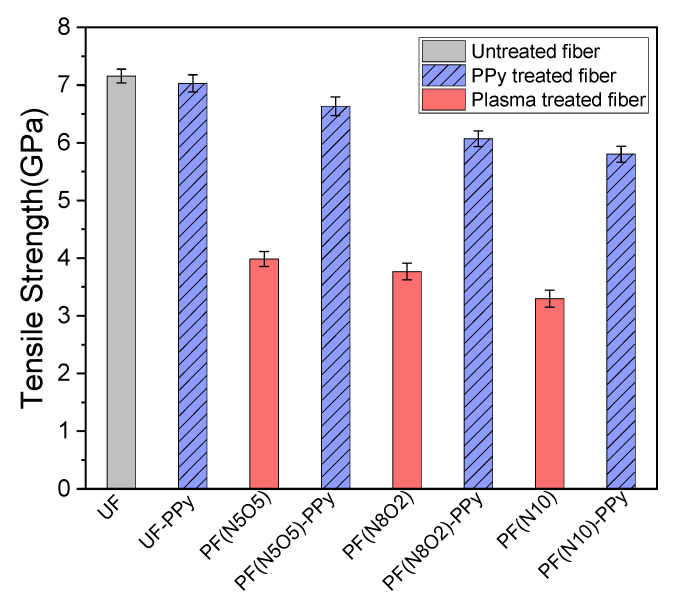
Tensile test results.

Figure 5 shows the variation of the monofilament tensile strength in the different treatment processes. Among them, the single-filament tensile strength of the untreated fiber sample (UF) was the largest. It indicated that any surface pre-treatment process would decrease the tensile strength of the single filament. Compared to the monofilament tensile strength, it decreased significantly after the plasma treatment. The tensile strength of PF(N5O5), PF(N8O2), and PF(N10) decreased from 7.156 GPa to 3.985 GPa, 3.768 GPa, and 3.298 GPa, respectively. After the PPy grafting, the tensile strength of PF(N5O5), PF(N8O2), and PF(N10) was enhanced to 6.633 GPa, 6.071 GPa, and 5.803 GPa, respectively. It can be seen that the collaborative treatment process appropriately compensated for the excessive fiber strength damage caused by a single treatment method.

The PF(N5O5)-PPy only reduced the tensile strength of the original UF monofilament by 7%. After the plasma was treated, cracks, pits, and grooves appeared on the surface of the UHMWPE fibers. Although these defects reduced the tensile strength of the monofilament, they increased the interface performance between the fiber and matrix [24]. After the PPy grafting process, the microscopic defects on the monofilament surface could be repaired to a certain extent. Using the combination of the two methods, a significant enhancement of the IFSS of the UHMWPE fiber/epoxy could be achieved without dramatically sacrificing the tensile strength of the fiber.

The interior and surface of the fiber monofilament introduced certain micro-defects during its production and surface treatment. The distribution of these defects affected the microstructure and tensile strength of the fiber monofilament. On the contrary, the fiber statistical law of the monofilament tensile strength also reflected the law of the influence of the different manufacturing and surface treatment processes on its microstructure. Since the UHMWPE fiber is a fragile material and follows the weakest chain theory, its tensile strength conforms to the two-parameter Weibull distribution function [25,26,27]. Therefore, the stress σ can be represented as a one-dimensional Weibull distribution function for the fracture probability, as shown in Equations (3) and (4).

(3) $$P\left(\sigma \right)=1-{\left[1-F\left(\sigma \right)\right]}^{N}$$

(4) $$F\left(\sigma \right)=1-\exp\left[-{\left(\frac{\sigma }{\sigma_{0}}\right)}^{m}\right]$$

where *P*(*σ*) illustrates the fracture probability of *N* defects under stress *σ*; *F*(*σ*) represents the tensile stress, which is the cumulative probability distribution function corresponding to σ; *m* is the shape parameter of the fiber, also called the Weibull parameter; *σ*_0_ indicates the scale parameter under the corresponding test span; and *σ* shows the strength test value of the fiber. The failure probability *F* corresponds to a certain stress level and can be expressed using Equation (5).

(5) $$F=\frac{i}{N+1}$$

where *N* is the number of samples of the tested single fiber and *i* is the serial number of the strength data obtained from the test sorted from small to large.

In order to obtain the shape parameter *m* and scale parameter *σ*_0_ of the Weibull distribution function, two logarithms were taken on both sides of Equation (3) to obtain Equation (6).

(6) $$\ln\ln\left(\frac{1}{1-F}\right)=m\ln\sigma -m\ln\sigma_{0}$$

**Figure 6:**
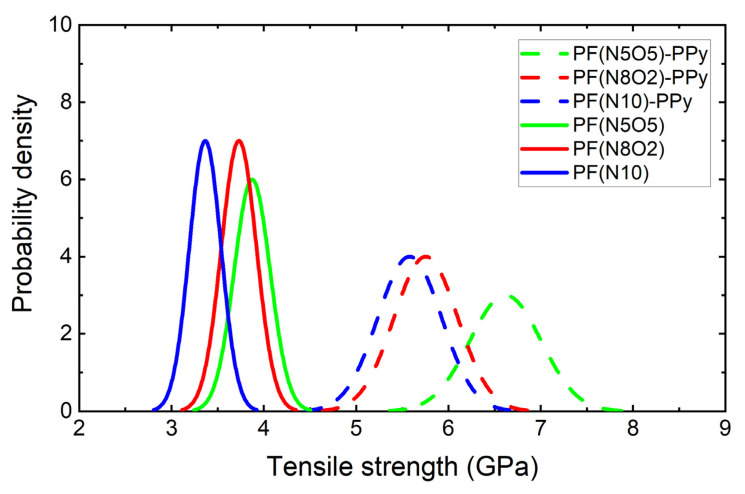
Fiber strength density distribution.

It can be seen from Equation (6) that *lnσ* and lnln(1/(1 − *F*)) have a linear relationship. Here, a scatter plot of *lnσ* and lnln(1/(1 − *F*)), a one-variable linear regression equation and a Weibull distribution double logarithmic graph, were drawn and analyzed. Among them, the shape parameters of the Weibull equation were obtained by calculating the slope of the straight line equation, and the scale parameters of the Weibull equation were obtained using the intercept. The relevant result is shown in Figure 6 and the parameter calculation result is shown in Table 2.

**Figure 7:**
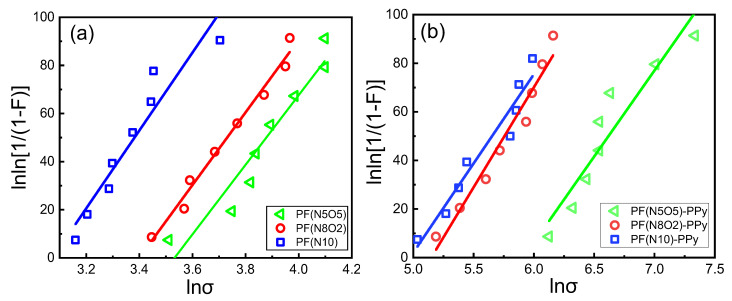
Weibull double logarithmic diagram: (**a**) plasma treatment; (**b**) plasma treatment and PPy grafting.

The shape parameter m is a characterization of the dispersion of the strength of the fiber monofilament. The larger the m value, the smaller the dispersion of the strength. The scale parameter σ_0_ was the size of the tensile strength. With the larger values of *σ*_0_, the values of the strength of the fiber increased. A more intuitive view of the data can be obtained from Figure 7, which shows that the scale parameters of PF(N5O5) and PF(N5O5)-PPy samples were significantly larger. This indicates that the tensile strength of the fiber monofilaments was higher, which was consistent with the findings presented in Figure 5 from a previous study. After the plasma–PPy synergistic treatment of the fiber monofilament, the fiber defects were repaired, improving the tensile strength of the monofilament.

**Figure 8:**
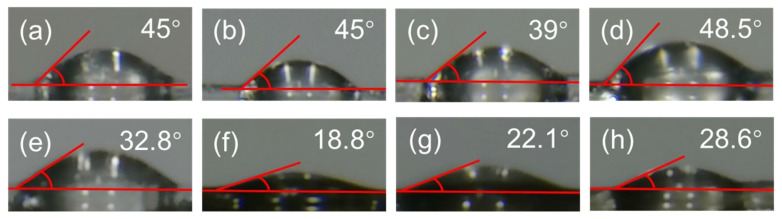
Contact angle of the fibers: (**a**) UF; (**b**) PF(N5O5); (**c**) PF(N8O2); (**d**) PF(N10); (**e**) UF-PPy; (**f**) PF(N5O5)-PPy; (**g**) PF(N8O2)-PPy; (**h**) PF(N10)-PPy.

In the traditional sense, the strength distribution of the UHMWPE fibers can be described by the standard Weibull distribution function and as a normal Gaussian distribution as well [28]. The Gaussian distribution probability of the fiber strength density is shown in Figure 6. Overall, the distribution probability density function image curve peaks of the PF(N10), PF(N10)-PPy, and PF(N8O2)-PPy samples were lower, and the curve width was relatively wide, while the curve peaks in the images of the other three fiber samples were higher and the curve width was narrower. This shows that the dispersion of the former three samples was relatively large, while the dispersion of the latter three samples was relatively small. Moreover, the PF(N505)-PPy, PF(N8O2)-PPy, and PF(N10)-PPy images were shifted to the right compared to the other three fiber strength density distributions that only underwent the plasma treatment, indicating that the fiber strength after the PPy grafting was increased. Compared to Figure 8, the results were consistent. The image of the PF(N10) fiber sample was to the right of the PF(N5O5) and PF(N8O2) images, which also showed that the tensile strength was higher than the other two fiber samples.

### 3.3. Effect of the Surface Treatment on the UHMWPE Fibers

Figure 8 shows the test results of the contact angle of the surface of the fibers before and after the surface modification treatment. As shown in Figure 8a–d, the contact angle of PF(N5O5) was 45°, which was similar to the UF. However, PF(N8O2) had the smallest angle 39° (Figure 8c). This showed that for the plasma treatment, a larger degree of etching led to a smaller contact angle and a better wetting performance, and the formation of the -C=O, -OH and -COOH functional groups with plasma treatment were more hydrophilic than the original fabric [29]. In Figure 8d, it can be seen that the contact angle of the PF(N10) was 48.5°, which was the largest contact angle in these samples. Since the pure nitrogen etching degree was very high, the fiber received too much damage and the performance was reduced. Figure 8e–h shows the contact angles on the basis of (a) to (d) using PPy grafting. It can be seen that the fiber after PPy grafting had a smaller contact angle and a better wettability than the untreated fiber. For PF(N5O5)-PPy, PF(N8O2)-PPy, and PF(N10)-PPy, the contact angles of the fibers grafted with PPy were obviously different. PF(N5O5)-PPy had the smallest contact angle, 18.8°, which was 58.2% smaller than that of the untreated fibers. Hence, PF(N5O5)-PPy showed the best wetting performance, which enhanced the composite performance between the UHMWPE fiber and the epoxy resin.

**Figure 9:**
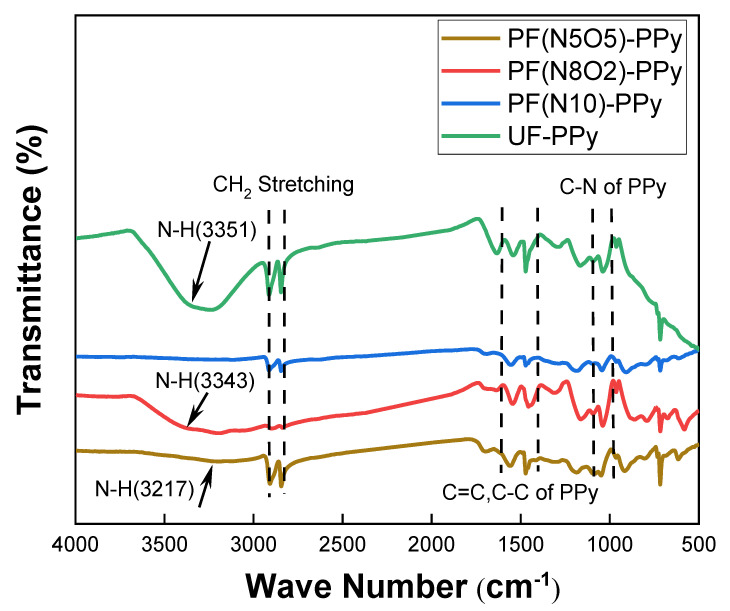
FTIR after PPy grafting.

As shown in Figure 9, alterations in the molecular groups were observed using FTIR spectroscopy. The plasma treatment used different types of plasma gases to generate free radicals on the fabric’s surface by impacting surface ions and causing polymer chain scission [30]. During the plasma process, groups such as -C=O, -OH, and -COOH were mainly found, which were easy to combine with the N-H of the PPy, making it more hydrophilic than the original fabric [29,31].

Figure 9 shows an infrared spectrum of the PPy grafting after the plasma treatment. It can be seen that two absorption peaks of the benzene ring appeared at 1600 cm^−1^ and 1510 cm^−1^. The strong absorption peak at 1037 cm^−1^ was the stretching vibration of the -CO bond and the wide frequency band at 3040–3400 cm^−1^. The absorption peak of N-H from 3351 cm^−1^ decreased to a lower wavenumber 3217 cm^−1^ (PF(N5O5)-PPy), which indicated that hydrogen bonds formed between the PPy and the UHMWPE interface. Furthermore, the stretching vibration of CH_2_ was near the absorption peaks of 2914 cm^−1^ and 2845 cm^−1^, the absorption peaks were in the range of 1200 cm^−1^~1300 cm^−1^ belonged to the -CN stretching vibration, and the absorption peaks of 1536 cm^−1^ and 1406 cm^−1^ belonged to the -C=C and -C-C stretching vibrations, which was the characteristic absorption peak of the pure PPy. In Figure 9, the characteristic absorption peaks of the PPy can be observed simultaneously, indicating that the PPy was successfully attached and grafted onto the surface of the UHMWPE fibers.

**Figure 10:**
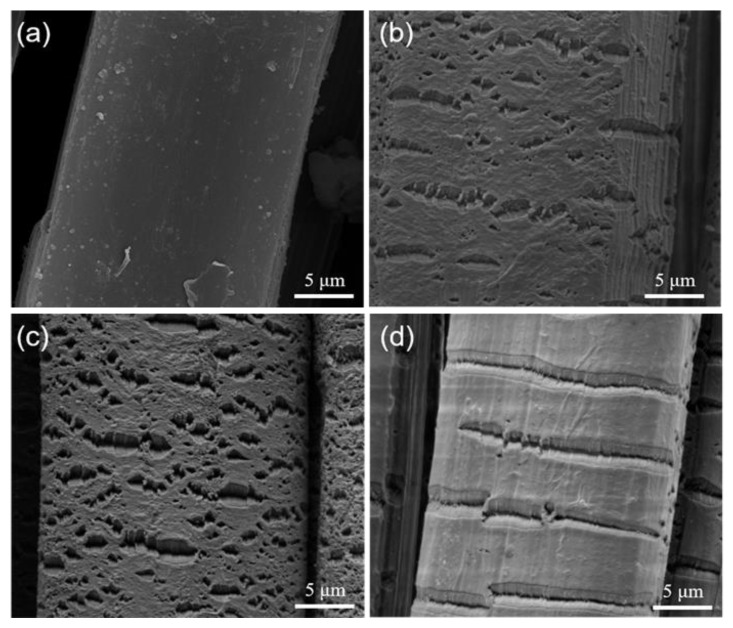
Surface micromorphology of the UHMWPE fibers before and after the plasma treatment: (**a**) UF; (**b**) PF(N5O5); (**c**) PF(N8O2); (**d**) PF(N10).

Figure 10 shows the micromorphology of the UHMWPE fibers through the plasma treatment and the untreated fibers. Figure 10a shows the surface micromorphology of the untreated UHMWPE fibers. Compared to Figure 10b, it can be seen that the pits on the surface of the fiber show an even distribution and small pit-like etching with a ratio of 5:5 for the oxygen and nitrogen mixture in the plasma treatment. Figure 10c shows the microstructure when the proportion of the plasma treatment atmosphere changed from 5:5 to 8:2. Continuous and dense indentations can be clearly seen, and the degree of the indentations became deeper compared to the fiber PF(N5O5). When the atmosphere became pure nitrogen, shown in Figure 10d, regular cracks and longer grooves were arranged on the fiber surface. It was easy to note that with an increasing percentage of nitrogen in the plasma treatment atmosphere, the etching became obvious and denser, which indicated that the use of inert gas for the plasma treatment had a strong etching effect. With the increasing oxygen ratio, the atmosphere became more active and caused a lower degree of etching. Thus, pure nitrogen had the deepest etching degree [32]. The generation of pits increased the friction on the fiber surface to better attach PPy, but also reduced the tensile property of the fiber. Figure 10 shows the uniform pits and effectively grafted PPy, which greatly improved its shear properties while maintaining its tensile properties. Therefore, PF(N5O5) (Figure 10b) had a better effect on the PPy grafting.

**Figure 11:**
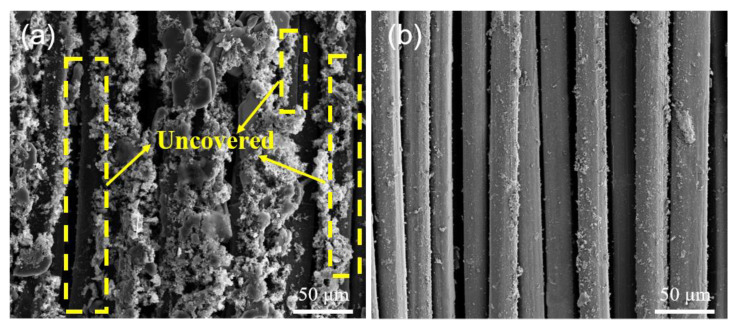
Surface micromorphology of the UHMWPE fibers after the plasma treatment and grafting of PPy: (**a**) UF-PPy; (**b**) PF(N10)-PPy.

Figure 11a shows the surface micromorphology of the untreated UHMWPE fibers grafted with PPy. The surface shows the agglomeration phenomena of PPy with an uneven distribution, and the part of the UHMWPE fibers was still uncovered by PPy. Figure 11b shows the surface micromorphology of the UHMWPE fiber after the plasma treatment and grafting of PPy. Compared to Figure 11a, that the fiber was fully covered by PPy with a smooth surface, and no pellets and impurities were found. This proves that PPy can be better grafted onto the fibers after the plasma treatment.

**Figure 12:**
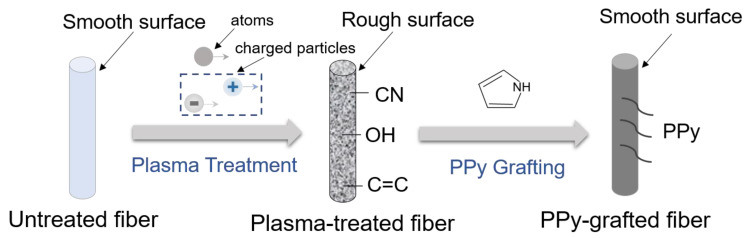
The illustration of the modification mechanism of the UHMWPE fiber.

Figure 12 shows the illustration of the modification mechanism of the UHMWPE fiber. In this study, the UHMWPE fiber was treated with plasma to increase the surface roughness. In addition, charged particles impacted the atoms or molecules in the gas during the glow discharge, which activated the surface tissue of the UHMWPE fiber and promoted the in-situ growth of PPy. In this process, PPy can be completely wrapped around the surface of the fiber, effectively filling the cracks during the plasma treatment. Hence, it can repair the damaged area of the fiber. Finally, the interfacial shear strength of the composites were greatly improved by means of a collaborative treatment. As shown in Table 3, combining the experimental results of microsphere debonding and filament stretching, the tensile strength of the plasma-treated fiber filaments generally decreased while the shear strength of the fiber/matrix interface increased. At the same time, compared to the untreated fibers, the tensile strength of the PF-N5O5 fiber was reduced by 7.3%, and the interfacial shear strength increased by 357%, compared to the methods from the other existing studies, in which the interfacial shear strength improved only by 26.6% or 84%, respectively [12,18]. A better improvement was obtained in this study. The results indicate that the growth of PPy was uneven and there was a phenomenon of stacking in some places, which could not be completely wrapped without the plasma treatment. However, after the plasma treatment, PPy was uniformly in-situ grown on the surface of each fiber, resulting in a smooth surface appearance. The synergistic treatment process of the UHMWPE fiber is shown in Figure 12.

## 4. Conclusions

In this work, a novel physical–chemical modification method was presented to improve the interfacial property of the UHMWPE fiber and epoxy resin. The UHMWPE fiber was successfully grafted with PPy for the first time after the plasma was treated in an atmosphere of mixed oxygen and nitrogen. It was found that the combined use of the plasma treatment and PPy in-situ grown on the fiber surface was an effective way to significantly improve the adhesion between the UHMWPE fibers and the epoxy resin. The results were expected to propose a surface modification technique for the UHMWPE fiber that would be effective, affordable, and environmentally friendly, hence providing opportunities for improving the mechanical characteristics of composites reinforced with UHMWPE fibers. The following conclusions can be obtained from this work.

After the plasma treatment, the UHMWPE fibers were etched with increased surface roughness. Following the PPy in-situ grafting, the surface of the fibers became smooth.

Through a combination of plasma treatment and in-situ grown PPy, the UHMWPE fiber exhibited an optimal modification effect with a balanced atmosphere mixture of O_2_ and N_2_, resulting in an IFSS of 15.75 MPa and tensile strength of 6.633 GPa.

In comparison to the pristine fibers, the UHMWPE fiber under the combination modification of the plasma treatment and in-situ grown PPy (equally mixed atmosphere) showed a good balance of interfacial performance and tensile strength. The IFSS was significantly enhanced by 357%, while the tensile strength was only slightly reduced by 7.3%.

The enhancement of the interfacial performance was attributed to the increased fiber surface roughness and in-situ grown groups, which improved the surface wettability between the UHMWPE fibers and the epoxy resins.

## Figures and Tables

**Table 1:** Sample specifications before and after the surface treatment.

Materials	Sample Code	Details of the Surface Treatment
Untreated fibers	UF	—
Fibers treated with plasma in a gas mixture	PF(N5O5)	Volume ratio of N_2_ to O_2_ = 5:5
	PF(N8O2)	Volume ratio of N_2_ to O_2_ = 8:2
	PF(N10)	Pure nitrogen
Fibers treated with PPy	UF-PPy	PPy treated on the basis of UF
	PF(N5O5)-PPy	PPy treated on the basis of PF(N5O5)
	PF(N8O2)-PPy	PPy treated on the basis of PF(N8O2)
	PF(N10)-PPy	PPy treated on the basis of pure nitrogen

**Table 2:** Equation parameters of the Weibull model.

No.	Materials	Scale Parameter Estimates ( $\sigma_{0}$ )	Shape Parameter Estimates (m)
1	PF(N5O5)-PPy	6.78	20.24
2	PF(N5O5)	3.97	22.4
3	PF(N8O2)-PPy	5.91	17.89
4	PF(N8O2)	3.82	21.51
5	PF(N10)-PPy	5.8	16.79
6	PF(N10)	3.45	27.06

**Table 3:** IFSS and single-fiber tensile strength of the untreated plasma and the PPy-treated fibers.

No.	Materials	IFSS (MPa)	Increment	Tensile Strength (GPa)	Decrement
1	UF	3.45	——	7.156	——
2	UF-PPy	7.21	109%	7.029	17.7%
3	PF(N5O5)	3.48	0.9%	3.985	44.3%
4	PF(N5O5)-PPy	15.75	357%	6.633	7.3%
5	PF(N8O2)	4.55	32%	3.768	47.3%
6	PF(N8O2)-PPy	10.08	192%	6.071	15.2%
7	PF(N10)	2.25	——	3.298	53.9%
8	PF(N10)-PPy	8.08	134%	5.803	18.9%

## Data Availability

The data that support the findings of this study are available upon request from the corresponding author upon reasonable request.
